# The spaces and times of community farming

**DOI:** 10.1007/s10460-016-9717-0

**Published:** 2016-08-15

**Authors:** Pingyang Liu, Paul Gilchrist, Becky Taylor, Neil Ravenscroft

**Affiliations:** 1 Department of Environmental Science and Engineering, Fudan University, 220 Handan Road, Yangpu, Shanghai, 200433 People’s Republic of China; 2 School of Environment and Technology, University of Brighton, Brighton, BN2 4GJ UK; 3 Plumpton College, Ditchling Road, Nr Lewes, East Sussex BN7 3AE UK

**Keywords:** Community farming, Everyday life, Therapeutic landscapes

## Abstract

This paper uses a multiple case study approach to researching people’s everyday lives and experiences of six community farms and gardens in diverse settings in China and England. We argue that collective understandings of community are bound up in everyday action in particular spaces and times. Successful community farms and gardens are those that are able to provide suitable spaces and times for these actions so that their members can enjoy multiple benefit streams. These benefits are largely universal: in very different situations in both England and China, CSA members make strong connections with the land, the farmers and other members, even in cases where they rarely visit the farms and gardens. This suggests that community farming and gardening initiatives possess multi-dimensional transformational potential. Not only do they offer a buffer against industrialised and remote food systems, but they also represent therapeutic landscapes valued by those who have experienced time spent at or in connection with them. Our findings indicate that—regardless of location or cultural context—these benefits are durable, so that people who have been engaged in multiple activities at a community farm or garden continue to enjoy these benefits long after most of their engagement has ceased.

## Introduction

It is common for papers on urban and Community Supported Agriculture (CSA) to extol the virtues of the community aspects of CSA (see, for example, Wells and Gradwell 2001; Watts et al. 2005; Firth et al. 2011; Flora and Bregendahl 2012; Obach and Tobin 2014), in many cases arguing that they are as least as important as the food that is produced (Amsden and McEntee 2011; McIver and Hale 2015). For others, including Shi et al. (2011) and Wittman (2009), new forms of agrarian or ecological citizenship have the therapeutic potential to address the ills of agribusiness (Schneider 2015), promote care of the self (Jarosz 2011; Ravenscroft et al. 2013) and heal what Wittman (2009) characterises as the metabolic rift that has opened between society and nature. Indeed, it is widely claimed that an ethic of care, allied to a response to food scares (Sempik and Aldridge 2006; Jarosz 2008, 2011; Shi et al. 2011; Qu and Jiao 2013; Si et al. 2015) is a strong motive for many people to get involved in CSA and other such projects.

Yet, there are counter-arguments emerging that question whether these claims can be substantiated (De Lind 1998; Guthman 2008; Chen 2015a; McIver and Hale 2015). In their recent work, for example, Pole and Gray (2013, p. 97) suggest that the community aspects of CSA may now—for many people—be “ancillary” to securing fresh, local and seasonal produce (which may well have associated health benefits). Galt et al. (2015) have similarly questioned who belongs to CSA and why, while in earlier work Galt (2013) also notes (following claims by Groh and McFadden 1997) how CSA farmers continue to subsidise CSA members through failing to take adequate compensation for their labour, a situation not dissimilar from that found in early CSAs in China (Shi et al. 2011; Chen 2015b). Indeed, with the availability of virtual platforms such as Taobao and Wechat in China, and Facebook in the West, it is apparent that spatial detachment to growing communities has become a customary practice as few CSA members need to visit the farms from which they secure their food meaning that, for many, CSA need be little more than a consumption decision (Carolan 2011; Chen 2015a).

While this may be so, a decline in spatial connection does not always mean a weakening of community ties. Many people value CSA for more than food without the necessity of visiting on a regular basis (see, for example, Jarosz 2011; Flora and Bregendahl 2012; Ravenscroft et al. 2013). However, we need richer ways of understanding this value in communities associated with food, given that current conceptualisations are too broad and contested to be helpful in identifying how individuals relate to each other and to the land and environment. Thus, we take a closer look at people’s experiences of community supported agriculture and associated community food projects, as a means of better understanding what it is that people value about the particular food communities to which they belong. This involves reflecting on people’s everyday practices when visiting CSAs (Day 2006) and the spaces and times in which they occur (O’Hara and Stagl 2001; Warde 2005; Shove 2009; Flora and Bregendahl 2012).

In their work, Flora and Bregendahl (2012, p. 332) argue that “place matters,” certainly to the extent that it reflects unique environments and contexts, as well as offering the possibility for social practices to take place. That these practices take place is a matter of time allocation for those involved, with the relative utility of different practices determining the time that is given to them (Shove 2009, p. 24). This resonates with our own experiences of food-growing communities in China and the UK where people consistently articulate their sense of community by references to activities that have taken place in specific spaces and times. Following Sennett (2012), we have also observed that these references are often bound up in memories that allow individuals to “place” themselves, spatially and temporally, in ways that help them to make sense of their attachment to their community and the benefits that they receive from the community. In this way, we feel, a community farm does not have to (or perhaps cannot) be known or understood in any over-arching sense, but instead becomes—partially and intimately—known by its persistent bugs or friendly animals, just as an allotment garden becomes known for the speed at which weeds seem to grow. For many people, this is the very stuff of community farming and gardening:

Autumn, with a lot of work in our CSA, gives me an opportunity to lament on my thoughts. On an October pick-up day, it is my turn to help with the distribution. I do this with joy and pleasure. I enjoy socializing with members, help them, I do it for good reasons, for the community, for the ideal of the CSA, something which is beyond my own interest. Every movement I do—taking out empty boxes to the car, cleaning the tables, sweeping the hallway of the school—is a meaningful, truly human activity for me. The small gesture of sweeping gives me a true experience. … This is not the same sweeping I do in my courtyard, or to be more precise, it is the same act but with a different meaning. And the meaning seems to be more important than the action itself (Kis 2014, p. 290).

It is these meanings that are also important to us because, as Kis observes, they constitute a connection with places and people that is deeply personal, in combining time (a day in autumn), space (a school hallway) and activity (helping distribute food). She understands her connection in these terms; taking her turn, going beyond her own interest, doing things that are both minor daily chores and tasks imbued with meaning, countering the increasing speed of everyday life (Virilio 1977; Shove 2009). Many records of community supported agriculture contain similar details (see, for example, Groh and McFadden 1997; Ravenscroft et al. 2013), often with overt reference to the social, pedagogic and therapeutic potential of such institutions (Wells and Gladwell 2001; Jarosz 2011; Ravenscroft et al. 2012). Yet we need equally to recognise that not all CSA members experience this (DeLind 1998, 2011), nor necessarily want it, certainly in terms of a totalizing notion of community (Mount 2012; Galt 2013; Si et al. 2015). Following work by Henderson and Van En (2007), it may be that some kinds of farms or CSAs facilitate therapeutic practices of community-building while others do not, just as some people seek community and others do not. As Mount (2012) has argued, the key point should be to recognize diversity in CSA practices and consumer intentions, which raises the question of how this corresponds to the differences that we see in members’ motivations and practices.

Thus, we want to address an apparent gap in the literature by focusing attention on the factors that seem to foster community-building in CSA membership and practice, concentrating in particular on the claims made about the therapeutic potential of CSA, in terms of the physical environment (Morrice 1979; Gesler 1992, 1993) and “social climate” (Moos 1997) of CSA. Informed by Flora and Bregendahl’s (2012) work on balancing the flow of community capitals within large CSAs, we see this as particularly significant in opening up new understandings of the relationships between people’s consumption decisions and the wider benefits that they gain from practicing community through CSA and other forms of food communities. We have chosen to focus on China and the UK because their engagement with, and experience of, CSA is at very different stages of development. In the UK, CSA is an established, if still relatively novel, form of agricultural organisation that is largely counter-cultural in reifying small farms and local food (Saltmarsh et al. 2011; Ravenscroft et al. 2012, 2013). This is not the case in China, where CSA is very much in the start-up phase and is utterly dominated by consumers (Si et al. 2015) despite there being increasing recognition of the need to protect small farms and traditional farmers (Schneider 2015; Liu et al. 2016).

We start by reviewing the literature that refers to the times and spaces of community, to develop a suitable research question and methodological approach to data generation. Using case studies of six community farming initiatives in China and the UK (not all formally CSAs, but all engaging communities in or with food production), we seek to argue not only that certain activities anchored in particular spaces and times give meaning to community membership, but also that the spaces and times inhabited by food communities can be, and in many cases are, avowedly therapeutic. Our findings indicate strongly that while the symbolism of communities supporting agriculture is not lost on most of those involved, it is individual bodily experience of a farm or garden that impacts at a deeper psychological—and thus therapeutic—level. We conclude by suggesting that the identification of factors associated with community constitutes a new insight into local food initiatives. However, we recognise that this needs to be read alongside Mount’s (2012) warning that even the most ardent CSA members tend to adopt hybrid approaches to securing food, thus tempering any totalising claims about the utility—to individuals and society—of CSA.

### Spaces and times as organising concepts

According to the community studies literature, there has been something of a social “turn” towards the idea of community as an antidote to the flux and uncertainty of contemporary life (Kuecker et al. 2011). While some authors associate this with a sense of loss, recovery and continuation of tradition (Delanty 2010), others construct contemporary communities as complex, dynamic, contingent and networked approaches to maintaining a sense of “local” and “connectedness” within an increasingly impersonal and globalising world (Crow et al. 2002; Gilchrist 2009). In addressing this latter construct, Block (2008) argues that people increasingly “experience” community rather than belong to it. This means that choices are made and remade as people’s demands, needs and circumstances change, with the idea of “community” increasingly reflecting moments of shared interest or motivation rather than long-term and stable sites and practice and representations of identity.

Within this new understanding of community, ideas of space and time shift, from structuring factors such as the geographical territory of the neighbourhood and the clock time that signals work and play (Crow et al. 2002), to a more fluid and dynamic construction associated with the performance of social relationships, such as those found in CSAs (Warde 2005; Pollan 2008; Carolan 2011; Bastian 2014; Ghose and Pettygrove 2014). This is well illustrated by the work of Flora and Bregendahl (2012), on the application of the Community Capitals Framework to collaborative Community Supported Agriculture. In this study, the authors show how successful and sustainable collaborative CSAs work to create a balance of interests (capital flows) between producers and consumers that encourages continuity, precisely because “collaborative CSAs are dynamic organizations with flexible boundaries and dynamic relationships that form and reform over time” (Flora and Bregendahl 2012, p. 344).

As the work by Flora and Bregendahl (2012) suggests, the performance of contemporary collaborative CSAs is closely related to the processes of “… social production … and bodily deployment …” (Low 2008, p. 25), at least to the extent that the deployment of the capital flows is a social practice performed by the producers and consumers. For Lefebvre (1991), this means that such practices are part of the performance of everyday life and that “community” is, consequently, part of this performance rather than constituting part of the structure of the performance. According to Giddens (1984) and Low (2008), the social practices of everyday life are largely habitual, bringing with them a sense of routinized security and belonging, such that minor activities undertaken at different times in familiar spaces become emblematic of what Giddens (1984, p. 24) has referred to as “ontological security”. This means that people:

…. do not have to think long about what way they take, where they situate themselves, how they store goods, and how they connect things and people. They have developed a set of habit-determined activities that helps them organize their day-to-day life. Even when day-to-day practices are disrupted or when a situation is novel, it is possible to fall back on routines (Low 2008, p. 36).

This is precisely what Kis (2014) describes in the quote given in the Introduction: the habitual practice of community ordered by the seasonal rhythm of farming. As Kis observes, she performs many of the same activities as at home, but they have a different meaning because of the space that she inhabits at that moment and the time that she devotes to the activity; she understands—and celebrates—community through the space, the time and the actions that she takes. For her, the distribution of community capitals is such that she feels able and willing to perform community, and associates this performance with the activities that she undertakes. Community membership is thus for her a performative act: she participates because she is motivated and able to do so.

The implication is that the everyday practices associated with the use of space and time are a primary way in which we understand and experience community. It is within this context that we decide—almost on a daily basis—whether our current practices continue to be appropriate to the type of association that we wish to have with the community. While not suggesting that we can know a community through the spaces and times in which it is practiced, or experienced, this does suggest that a study of the spatial and temporal practices of community may give us new insights into the factors that tend to be emblematic of successful, enduring and therapeutic communities. Our research question is thus: to what extent do the intimate experiences of community—situated in particular spaces and times in very different locations and cultures—reveal factors that are significant in sustaining CSAs and other similar agri-food communities?

## Methods and procedures

In addressing this research question, we are thus seeking evidence about the ways in which people refer and relate to community farms and gardens across a range of locations, cultures and contexts. In order to do this, we have chosen to compare six very different farms and gardens in China and the UK, using a constructivist approach to data generation that focused on how CSA members in each of these situations make meaning from their bodily experiences. This means that the individual is of primary concern, but within a spatial and temporal context that involves interaction with other people (Laukner et al. 2012). This interactional focus meant that data need to be generated from place-based real-world communities (Yin 2009), with these communities forming the unit of analysis. The multiple case study method used for the data generation involved selecting cases according to two criteria: that they were exemplars of their type; and that they reflected two very different societies in which community food initiatives are at different stages of development (the UK and China). As Hartley (2004) has observed, the question of how many cases to select continues to exercise case study research. The decision was taken in this research to use the established community food sector in the UK as the guide, with three distinct types of organisation identified and represented: the large community farm; the small city farm; and the shared allotment garden. While it is not yet possible to delineate comparable organisations on this basis in China, three contrasting farms were chosen: one developing a short but sustainable supply chain (with some similarity to the large community farm in the UK); one with a strong education base (similar to the UK city farm); and a small farm still run in a fairly traditional manner (with similarities to the allotment garden in the UK).

While there are several approaches to generating data within a constructivist paradigm, initial meetings with the communities found that many members wanted to get involved in designing, as well as participating, in the research process. Indeed, for some of them, participation in the research was conditional on being involved in a co-design process. We embraced this request, and co-developed a participatory framework (Pain et al. 2011; Gilchrist et al. 2015) that facilitated the communities—each working with a designated member of the research team—in co-developing a suite of data generation methods that gave them insights into their communities. Although these methods varied between communities, as did the identity of those involved, all data generation methods included interviews and discussions that were facilitated by trained members of the respective communities. These were supplemented at some of the communities by document analysis and participant observation, again undertaken by members of the communities. All formal sessions were audio recorded and transcribed, with other materials and observations also reproduced in digital form.

Through this process, each community generated a digital file of material addressing the research question. Given the small amount of text, no coding or data management software was used, on the basis that it was possible to describe and understand the texts in terms of the meanings that people brought to them without the need for intervening technology (see Chowdhury 2015). The approach taken was thus to develop some pre-figured codes or categories, based on the literature and research question, which could be used to commence the analysis. The texts were then analysed by a “constant comparison” method (Glaser and Strauss 1967) in which the labels “space”, “time” and “activity” were attached to relevant sections of text that could then be retrieved by category. This was very much a circular process of moving between data and concept, labelling elements of the data in order to build a picture for theoretical elaboration and sense checking that the pre-figured categories were suitable for the analysis.

To this extent the method used was a hybrid between conventional approaches to axial coding and Glaser and Laudel’s (2013) approach to qualitative content analysis. By constantly comparing the data match to existing knowledge and emergent themes, the team was able to escalate their understanding from the raw text files to a picture that informed the research. Through this process, the “activity” category label was found to be related strongly with the “time” category, which allowed them to be combined. However, “meanings” emerged as a significant theme and the texts were recoded accordingly, using the categories of: spaces; time/activities; and meanings. In many cases passages of text combined all three categories, as people sought to locate their thoughts and meanings both spatially and temporally. While maintaining the categorisation of individual phrases and sentences, the integrity of texts with multiple categories was maintained, to ensure that the relationship between spaces, times and meanings was not lost. An example of this is given in Table 1.

**Table 1:** An example of category labels attached to text

Text relating to space (italics); time/activity (underline); meaning (bold) “There were not that many workdays and they were often fairly haphazard and often not well attended. They have taken on a mythology—but they had lived their day very soon. *Potatoes and leeks were the main things, planting, weeding, harvesting.* *I remember weeding as tough… (it was) all too much for people who were only used to gardening.* Truth is that this was a relatively short-lived phenomenon, but people loved it at the time, being with others and getting your hands in the soil. Working hard, aching back, then stopping for a break*…* But numbers dwindled; it was hard to ensure that there was meaningful work to be done … it got in the way of farming (ha ha)*…*”	

All of the UK records have been anonymised. In some cases, the quotes are attributed to an individual, in which case that person is represented by an initial (which is not necessarily related to the person’s given or preferred name). In others, no attribution has been made, in deference to the particular community involved. Table 2 contains the details of the cases and the evidence generated for each.

**Table 2:** Description of the case studies

Name	Type of Farm	Location	Sources of Evidence
Tablehurst Community Farm, East Sussex, UK	Large community-owned social enterprise with substantial retail and catering outlets	Rural location adjacent to a large village, with good road access to several large settlements, including South London	Documents (reports, newsletters, newspaper cuttings); individual and group interviews; a whole-community ‘memory day’
Miu’er Farm	Small organic farm with varied types of community engagement including vegetable production & sales, youth education and opportunities for people to experience agriculture	Rural location, about 25 km from a densely populated urban area and 60 km from Central Shanghai. No localized markets for its produce	Individual in depth interviews, observations; Documents (meeting records, newsletters)
Spitalfields City Farm, Borough of Tower Hamlets, Central London, UK	Small city farm concentrating on education and health interventions for the local population	A small site located in a relatively poor and densely-populated city-centre neighbourhood in London, UK	Documents (reports, newsletters, newspaper cuttings); individual and group interviews; a collaborative quilt-making project
Xin’gen Farm	Small organic farm concentrating on food production and agriculture experiences for visitors	Chongming County, Shanghai, China. Relatively remote rural location, about 100 km from the city centre	Documents (Group Meeting records, newsletters)
Young Women’s Allotment Project, Manchester, UK	A small allotment garden run by youth workers with volunteer young women	A single plot in a publicly-owned allotment garden in a suburban location in south Manchester, UK	Individual and group interviews, observation, two video films
Mengtian Farm	Small organic CSA with stable membership, concentrating on food production	Chongming County, Shanghai, China. Relatively remote rural location, about 70 km from the city centre	Individual in depth interviews and observations; Documents (Group Meeting records, newsletters)

### The role of space and time in locating community

For many, in all the communities, the core meaning of the land is as a space that is theirs, where they can simply dwell, or can undertake fulfilling activities in the companionship of like-minded people. One member of the Tablehurst community, who worked in London, said that the farm “… connects me with the ground and gives balance to my life” (male, age 40). For another member of the community, “… the farm is an oasis in the midst of madness” (male, age 64). One of the founder members of the community went further, in claiming that “I always make sure [that] I have time to help out with the lambs in the spring. It’s an extraordinary experience … there’s a sense of guardianship over the ewes” (male, aged 35). In all these cases the distinction is made between a life external to the farm and the experience of being on the farm. Similar stories were told in China, by the farmer at Xin’gen Farm:

I didn’t like farming at first … [but] once I … experienced natural farming … It felt so different, so relaxed, even after I returned to work on Monday—Monday used to be a tough day for me. One time we harvested barley in the field, my whole family was really excited. Afterwards, with more and more consideration for food security, I decided to quit my job and to do organic farming myself. (Hou, Xin’gen Farm)

While Hou’s decision to leave her job may be more extreme than most, her observation about farm work balancing her off-farm life is redolent of many similar experiences, such as those of the Manchester women, whose allotment was experienced as a “safe” space where they could relax and unwind from the stresses of other parts of their lives. Typical of this is the following exchange:

A: I think it makes me a lot happier when I go to do other things. Like it’s … a thing at the end of the week …. It’s fine, I can do all of this hard work [away from the allotment] ‘cause I’ll get to spend 4 h on the allotment on some days.

B: It’s very therapeutic. So like weeding and weeding [all laugh] and yes, more weeding [all laugh] and, you know, just kind of just getting really stuck in. I think it gives you that head space where, you know, you’re able to kind of relieve maybe some kind of troubles and then you come out feeling refreshed, renewed and ready to carry on.

(Two young women in conversation, in response to a prompt in a focus group about what about the allotment makes them happy)

Another of the Chinese farmers described a similar experience:

People come to our farm not only for food … They come for the feeling of happiness, the happiness of experiencing nature, of learning something unknown before, such as knowledge of nature and farming. It’s the special service that keeps people supporting the farm. (Kang, Miu’er Farm)

For others, such as a young woman at Spitalfields City Farm, the association is with the animals, which she describes as her family: “… I do feel a strong bond to the animals … they are like my kids … if I am stressed out I go and cuddle a goat.” Others—at Tablehurst Farm—describe the early days of harvesting leeks, washing them in cold water, packing them and sliding the heavy crates over the muddy field. Another woman, also with reference to Spitalfields City Farm, observes:

We’ve cooked on a camp fire and made our own pizza dough and picked the vegetables and cooked them. People come for all kinds of reasons: there is someone who comes because their job is high powered and they just want a contrast; and someone else comes because they haven’t got a job; everybody finds it kind of restorative …  (woman, age 50).

The idea of the farm and garden as “restorative” comes through strongly in all of the case studies; people feel “differently” when they get to the farm or garden and many feel that they cannot imagine living in the area without it. Indeed, it becomes so much a part of the fabric of people’s lives that some, including a woman at Spitalfields City Farm, observe that they no longer feel the need to perform community:

It’s a nice place to almost not interact with each other; you don’t have to; you just sit and interact with the place and with nature … people don’t feel the pressure to chat, but they probably interact in a quieter way … (woman, age 34).

For others, the active performance of community is integral to their experience of the farms and gardens. For some this is about the social aspects (the dances, BBQs and rituals that occur periodically at most of the farms), while for others it is about shared work. Many people at Tablehurst Farm, for example, remember fondly the early days when “… there were lots of workdays …. And [1 year] the potatoes were harvested in one wonderful afternoon where lots of willing hands from the local community made it light work” (extract from the farm newsletter, 1996). Some years later, however, one of those who was involved reflected that:

… there were not that many [workdays] and they were often fairly haphazard and often not well attended. They have taken on a mythology—but they had lived their day very soon. Potatoes and leeks were the main things: planting, weeding, harvesting. I remember weeding as tough—50 rows, 200 yards long; it was all too much for people who were only used to gardening. Truth is that this was a relatively short-lived phenomenon, but people loved it at the time, being with others and getting your hands in the soil. Working hard, aching back, then stopping for a break and lovely cake from [the farmers] … (male, age 45)

A similar—but contemporary—story is told by the farmer’s mother at Mengtian Farm, where the romanticism of organic farming is tempered by recognition that people have to undertake hard labour for others to enjoy the harvest:

The farming is really tough. We have to work on the fields all the year, from early in the morning to late in the evening… most of the work is weeding and catching worms by hand (Chen’s mother, Mengtian Farm).

She went on to comment on the fragility of their approach to farming, observing that all her hard work could come to nought if people decided to source their food elsewhere:

We now have about 100 members. But we don’t know if it will be 120 or 90 several days later. …some of the members only pay 4–5 deposits. … About 50 % are long term members, with deposits of 50–100 times. …Many buyers shift from farms one after another (Chen’s Mother, Mengtian Farm).

The “deposits” referred to in this quote are the sums of money that members of many Shanghai CSAs pay in advance for produce. These payments are normally made via China’s biggest ecommerce website (Taobao) and, in effect, “reserve” a certain amount of produce for that member for the coming week. Those who are only willing to commit to small numbers of deposits at a time are those who are unlikely to remain loyal to a CSA over time. This is the worry for many small scale CSA farmers, who have few other opportunities to sell their produce.

Despite this type of uncertainty—more common in the Chinese than the UK case studies—most farmers and gardeners feel that the value they get from their work extends beyond the growing and sale of produce. Indeed, the Manchester women (and some others, particularly at Spitalfields City Farm), describe their allotment as their “therapy space”—where they can arrive feeling stressed and leave several hours later feeling more at peace. They also observed how it upset them that some people had not had the opportunity to experience the liberation of being in their own outdoor space. Some women related the allotment to other aspects of their health, observing that they eat better because they have grown the food, and that they have lost weight and are fitter, partly as a result of the gardening and partly as a result of other activities, such as cycling to and from the allotment. This is also the case at Tablehurst, where several parents described the security that they feel knowing that their children are eating good nutritious food, and in China, where Kang described the health benefits for those working on the farm:

People working [on the] farm are all local villagers over 55 years old. …They spent most of their life working in the fields, and it’s not easy for them to stop. They like the opportunity of working [on the] farm, as it’s not so heavy work and provides another source of income for them. Most of them [also] treat this job as exercise, and they are really in good health. It’s just like what they used to do. (Kang, Miu’er Farm)

For others, there is also a learning element to being part of a community food project, as described by one young Manchester woman with little previous experience of gardening:

I think it makes you feel more connected to kind of the land and knowing the stuff that you can bring out into everyday life so you can share that knowledge. And, you know, refer to it and just different work, you know, with family, with friends and things like that… (young woman, Manchester)

This is also highlighted as significant at Mengtian Farm, where the local people who work on the farm experience and learn about organic techniques that they can apply to their own gardens and allotments:

Those working in the field are mainly local women over 50 years old. Most of their job is weeding. They are quite happy when they are in the fields, with much chatting. … And sometimes I join them. … they also learn from our farm. For example, most of them have given up using chemicals fertilizers and pesticides, instead removing bugs from the crops by hand (Chen’s Mother, Mengtian Farm).

For some, the work of community farming and gardening is counter-cultural, particularly for women undertaking work that is conventionally associated with men and heteronormativity (Jarosz 2011; Moore et al. 2014). This was a strong theme in Manchester—that they had learned how to succeed at something that is not viewed as “women’s work” and that men had not been involved, and were not needed, at all. Indeed, some of the young women spoke of their mixed feelings about even inviting a group of men to visit the allotment: they had enjoyed showing what they had achieved, but they were also glad when the men had gone and the space was theirs again. The following conversation illustrates some examples of this understanding:

A: I think particularly because of wider society, women really have to fight for their own spaces and because a lot of public space and a lot of kind of, you know, things are either controlled by or emotionally controlled by men’s interests or what men choose is kind of the best thing to happen ….

B: I think the allotment’s quite good because it kind of actually sort of teaches you to be self-sufficient in a way and kind of, you know, it’s quite educational in that kind of sense. But then so it does detach from the normal stereotypical, you know, sit at home doing gardening.

C: I think as well though the allotment for me like kind of challenges the stereotypes that like we do all the digging, we build our own things, we do everything basically on the allotment. Whereas a few people I’ve spoken to in the last few weeks like, “Oh you can’t do that.” Well yeah I can, I’m just the same as everyone else, it doesn’t mean that I can’t do it. So from my perspective I think it does like hit head-on with some of them challenges, stereotypes.

(Three young women in conversation in a focus group, in response to a prompt about why the allotment is important to them)

What is clear from this is that the farms and gardens are “special” places where people can explore different ways and times of being and doing. This can be both contemplative (good places not to interact with others) and active (harvesting the leeks and challenging gender stereotypes). This suggests a knowing juxtaposition: of the field or allotment as being an apparently open and active environment that has been transformed into an intimate place in which to share private thoughts or moments. While being fully aware that the allotment is physically in a public place, surrounded by other allotments and housing, for example, the Manchester women felt that they could be private in a way that is not always possible elsewhere, even in spaces that have been designed to provide privacy. In essence, they suggest that the transitional nature of the allotment, as a form of public/private space (Moore et al. 2014), provides a respectful distance and security that can facilitate privacy, whereas everyone knows what a designated private space (such as in their youth centre) is used for, making the act of talking within that space essentially public, even if the content of the conversation remains private. The following extract, which is unashamedly long, illustrates this point, starting from an assertion about the sociability of being at the allotment:

A: It’s quite a social space as well. Like you kind of, I don’t know, like you get to know people [coughing] like maybe if you’re put with someone who say you don’t talk to a lot like… that you wouldn’t usually…

(facilitator): So is that different to other times that you spend with the young women; is it something different, or it is the same as at other times?

A: I suppose it’s the same a little bit and I’m kind of different because when we’re in a session at the Young Women’s Project we’re usually focussed on what we’re doing. But then say if you are like weeding, obviously you’re still focussed but it allows a bit more like freedom to talk. It’s not necessarily on a certain topic.

(facilitator): So you’re doing the practical activity of weeding but you don’t have to be talking about a particular topic?

B: Or weeding [all laugh].

(facilitator): So you can chat about your life? I’ve got you. So what does that mean to you? [Pause] Does it feel like a women’s allotment or a lesbian and bisexual women’s allotment?

A: Yeah.

C: I think ‘cause we know that it’s just young women that go on the allotment, you just feel more comfortable in just doing stuff and without feeling uncomfortable.

A: I think it makes it a lot easier to have those difficult conversations; at the allotment we’ve had some really difficult conversations but because we’re at the allotment and it’s women only it’s really easy to have those difficult conversations ‘cause you’re not really worried about what the young men would think if you had that conversation during [an official meeting] or something. And ‘cause it’s not in a set setting, it’s not in the Centre, it’s not [pause], it’s official but it’s not official.

(facilitator): Can you give us a kind of example of a theme?

A: Conversations about mental health. I’ve had a lot of those on the allotment with people. Or if you’ve just had a really tough week. It’s not really, well you don’t feel like you’re being analysed when you talk about it on the allotment. Whereas if you’re in the Centre it could seem that you were being analysed.

C: I think as well within the Centre people feel like other people can hear what they’re saying, whereas on the allotment it feels more open and it doesn’t feel like people are listening into your conversation. They probably aren’t anyway, but…

D: Yeah, that is true. Like in a really weird way because it’s obviously just a field essentially, like it’s easier to find a quiet corner there than it is at the Centre, which is weird, isn’t it?

E: Well I agree with what’s been said. Like it’s a lot easier to talk at the allotment and I think it’s nice that we have our own space, that is a women’s space and a lesbian and bisexual women’s space. Because I think when you’re there you feel you can talk more about the issues that we have in a less sort of closed setting really. And I think as well what it is, is because you like gardening you don’t really [pause], you sort of focus on that a bit more when you’re talking, so you talk easier. That’s what I find anyway.

(Five young women in conversation with the facilitator of a focus group)

This conversation evokes the idea of gardening at the allotment providing a suitable medium for facilitating “private” conversations: an activity such as weeding is both neutral and inconsequential to the subject matter, but it is widely understood as significant work that requires time to complete, and provides a “public” justification for the two people working and being together. This juxtaposition of intimacy in public (the public private of the allotment or field—see Moore et al. 2014) is both widely experienced and celebrated by the young women: it may be a field in suburban Manchester, but it is their lesbian and bisexual women’s field where they can take the time to perform the public and the private, the intimate and the mundane, in the ways, spaces and moments that they wish.

It is this same quality of relationship between people and land or animals that catalysed many of the earlier comments, about cuddling goats, helping with lambing and harvesting leeks. All these activities are simple and mundane; they are what people do in fleeting moments, day in and day out, in many contexts. But in the context of communities that farm and garden they take on a different level of significance, one that is related to the intimacy of shared practice. To this extent, the spaces and times referred to by those in all six case studies are deeply significant to individual people’s understanding of community and their intimate shared experiences of community. It is this intimacy that is key—that events happen in these spaces and times that have a meaning out of all proportion with the (minor) significance of the spaces and times themselves, thus meaning that weeding that row of carrots, at that time in suburban Manchester, is meaningful, just as surely as removing the worms from rice and barley in suburban Shanghai carries a sense of purpose and community for those involved.

### Experiencing community

There has been much recent academic and practitioner interest in the transformative potential of community food growing initiatives, in both urban and rural environments (Cox et al. 2008; Petherick 2010; Saltmarsh et al. 2011; Shi et al. 2011; Rioufol and Ravenscroft 2012; Ravenscroft et al. 2013). This is part of what has been referred to elsewhere as a cultural turn in farming and food production, away from intensive and industrialised farming towards what have become known as alternative food networks (Renting et al. 2003; Follett 2009; Wilson 2012; Si et al. 2015). There is certainly evidence of this in the research, particularly in the UK case studies. Yet, as Pole and Gray (2013) and Si et al. (2015) point out, “community” can be hard to locate, even for those who do participate in aspects of farming and gardening. This is most clearly the case in China, with many of those involved in the farms feeling isolated from the consumers, most of whom do no more than reserve their produce on-line. But there is also some indication of it in the UK, certainly that people experience the same community in very different ways.

As this research has found, especially in the UK, many of those who do become involved in community farms and gardens experience them as therapeutic learning environments through which they gain insights into themselves as well as life skills that they can transfer to other situations. This is particularly the case where there is the opportunity for practical work, such as gardening. Yet it is equally striking that, in other settings (suburban Shanghai), the same activities can somehow magnify the distance between the producers and consumers of the food. Context is therefore crucial: The Manchester women have their own space in which they work largely for themselves, in their own spare time. The Shanghai women (and some men) do the same work, but for others. While the Chinese farmers claim that their workers enjoy being in the fields, this is unlikely to be the same joy associated with the work in Manchester.

Quite apart from being new data about the embryonic and pioneer stages of community farming in Shanghai, what distinguishes the findings from other work that has also highlighted the therapeutic and developmental potential of community farming and gardening (see, for example, Jarosz 2008; Ravenscroft et al. 2012; Kis 2014) is the significance of specific spaces and times. Communities, whether involved in farming or other activities, are neither monolithic nor unified structures (Classens 2015), but are instead the sum of lots of people doing lots of things—separately and together—in lots of spaces over periods of time. Each of these actions, in each of these spaces, makes sense of community—for that person, at that moment. The momentary experience of these actions is critical in countering the ever-intensifying speed of everyday life (Virilio 1977; Redhead 2011). Preparing ground, planting, weeding and harvesting take time; time that could be devoted to other things but which—across all our case studies—is intentionally used for farming and gardening. This process of “slowing down” not only provides opportunity for discussion, but also for observation and contemplation of human and natural worlds (Ginn 2014). While not so apparent in the Chinese cases, it is still there—tales of “natural” work being fulfilling enough to give up paid employment, for example.

At the core of this spatial and temporal practice of community are remarkable events: individual and private communications and conversations taking place during mundane and repetitious activities in what are otherwise communal and semi-public spaces; spaces that are “just fields” rather than purpose-designed confessionals and consulting rooms. While rarely staged, these practices are far from random or without form. Rather, they reflect elements of social pedagogic practice in which mundane activities can unite people in common purpose (Stephens 2009; Eichsteller and Holthoff 2012; Berridge et al. 2011; Carolan 2011). Food growing provides a context to cultivate intimate relations which are co-produced through embodied and material practices involving nature. As Whatmore (2002, p. 162) writes:

… the rhythms and motions of… inter-corporeal practices configure spaces of connectivity between more-than-human lifeworlds; topologies of intimacy and affectivity that confound conventional cartographies of distance and proximity, and local and global scales.

In this case, interactions with nature through activities such as weeding provide a spatial and temporal medium for facilitating “private” conversations: the weeding is both neutral and inconsequential to the subject matter, but it is widely understood by the gardeners as significant work, and provides a justification for the two people working and being together. This juxtaposition of intimacy in public is both widely experienced and celebrated by many of those in all the communities. This does not mean that social relations are necessarily an outcome of social structures or community obligations but that they arise through a voluntary commitment to intimate shared practice in which individual benefits can accrue (Giddens 1992). This is so at all the case studies, whether relating to removing insects from vegetable plots in Shanghai, cuddling goats in East London, or washing and packing leeks in a field in rural England: the spatialized activities become the intangible commonality between people, providing them with an intimacy rarely experienced in other situations.

This finding is consistent with existing work on the characteristics of therapeutic environments (Morrice 1979; Gesler 1992, 1993). What is new is recognition of the ways in which community farms and gardens can provide the material context for symbolic mediation between people. Many of those who are involved in community farming and gardening understand—for themselves—that these spaces are symbolic of what they perceive to be therapeutic environments. This means that, for them, their farm community is about much more than food: it is about a broader and more encompassing understanding of well-being that extends beyond acts of food provisioning. Good and wholesome food that has health benefits can certainly be procured through CSA (Si et al. 2015). But intimate experience of the broader benefits of CSA and community food initiatives means experiencing the spaces and times in which community is performed through embodied and material practices. While this is most evident where there is physical engagement with the farm or garden, members of the Tablehurst community also seemed to experience the therapeutic environment simply by being at the farm, imbibing a sense of symbolic belonging. However, many of those making this connection were long-time community members who may well have “done their turn” with the physical activities sufficiently to feel connected even if they are no longer directly involved.

## Conclusions

In this paper we have sought to identify and explain some of the factors that are significant in sustaining food and farming communities in two very different contexts: established community farms and gardens in England; and pioneer community farms in China. The findings indicate—for both England and China—that space and time are significant in people’s experience of community; indeed, while the symbolism of communities supporting agriculture is not lost on most of those involved, it is individual bodily experience of a farm or garden that impacts at a deeper psychological level. As Carolan (2011, p. 58) has observed, “CSA encourages reflexive ethical reasoning.” Few of those involved in these case studies had any concrete notion of the communities to which they belong, but all could recount individual moments in time and space when they felt connected to the land and to those around them.

What was apparent in all of the case studies was a sense that the land, and activities relating to it, provided a healthy and therapeutic space and time in which those involved could say and do things that they would not say and do—in the same way—elsewhere. This was less evident for CSA members in China than in England, because there was less community involvement with the farms. While this might be explained by the relative youth of the Chinese CSAs, it is an important finding because the dominant message from the English case studies was that members get involved early in the life of the CSA and move on when they have fulfilled their ambitions. More research will be needed to establish where there is a different attitude to involvement in the Chinese farms, but the signs are that these CSAs are catalysing different forms of community that are less bodily engaged than their English counterparts.

In most of the case studies, the activities undertaken were relatively mundane, such as weeding, tending animals, harvesting and distributing food. But, for those involved, the activities were symbolic of their commitment to the farm or garden as well as being instrument in their own personal well-being. This is really an extension of Lefebvre’s (1991) argument that both space (land) and time are required to make bodily action possible—there is work to be done, that requires time to do it, if the farm or garden is to be productive—while the bodily action itself gives rise to outcomes beyond the production of food. In this case the dominant outcome relates to individual well-being—that those involved feel better as a result of their activity and the freedom that this activity gives them to do and say things that they would not ordinarily experience. This was the case even for the paid labour in China. There is also some evidence that involvement of this type can be thought of as a form of capital that can be stored and released in the future, such that the benefits continue to flow after the intense engagement has subsided. This is redolent of Flora and Bregendahl’s (2012) work on community capitals, in which they argue that people who gain multiple benefits (such as food, community and health in our case studies) are more likely to remain connected with their CSAs. This argument certainly fits the data, to the extent that the Chinese cases, where there is less engagement and community capital accumulation, have less stability and a higher turnover of members than is the case with the English case studies.

Consistency with earlier work is significant, given the different focus of the research. Indeed, while it is dangerous to generalise from these case studies of fairly evangelical people, it is clear that, for these people at least, active and participatory membership of community farming and gardening initiatives brings benefits well beyond access to fresh food. Anecdotally, for many of those involved the food is probably less important than the activity and symbolism of belonging. And, at the core of this therapeutic relationship is space—l and—and the moments in which it is filled with meaning, echoing Soja’s (1989) observation that the times of social practice actually constitute the space. In this way, we suggest that it is the very social and bodily practice of farming and gardening that constitutes the spaces and times of community—whether in terms of paid labour in China or volunteers in England.

In returning to our opening remarks and subsequent research question, it is apparent that while there is clearly diversity in CSA communities (Mount 2012), there are some factors that seem to be associated with vibrant and secure food communities. These factors align around fostering practices that help transform social relations from the relatively remote producer/customer relationship experienced in China to the more intimate shared practices found more commonly in the English studies. This is not to doubt that people can be committed to CSA without the need to visit and work at the farms, but it is to observe that the wider therapeutic benefits—to all involved—tend to be more evident where people are more spatially connected to their CSA. Indeed, for many of the participants in this research, a lesser level of spatial involvement may not affect the quality and teste of the food, but it may mean that some of the richer benefits that accrue by being on the land and growing together may never be realised.

## Abbreviations

CSA: Community supported agriculture
UK: United Kingdom
